# Supplementary material for: A Methodological Review of Mixed Methods Research in Palliative and End-of-Life Care (2014–2019)
Source: Int J Environ Res Public Health. 2020 May 29;17(11):3853. doi: 10.3390/ijerph17113853 (PMC7312170; doi:10.3390/ijerph17113853)
Supplement: Supplementary file 1 [file ijerph-17-03853-s001.zip › Supplementary Material/Supplementary_Material_1.docx]

**Supplementary material 1: Data extraction form**

| **Publication metadata** |
| --- |
| Article ID: |
| Publication year: |
| Author name/s: |
| Corresponding author affiliation: |
| Article title: |
| Journal name: |

| **Study purpose** |
| --- |
| Statements about the study purpose: |

| **Quantitative and qualitative components** |
| --- |
| Statements about the quantitative sampling: |
| Statements about the quantitative data collection: |
| Statements about the quantitative data analyses: |
| Statements about the qualitative sampling: |
| Statements about the qualitative data collection: |
| Statements about the qualitative data analyses: |

| **Mixed methods research component** |
| --- |
| **Description of mixed methods research** |
| Statements about the self-description of the study as mixed methods research: |
| Statements about key mixed methods literature cited *(include name of the reference)*: |
| **Justification for using mixed methods research** |
| Statements about the justification for using mixed methods research: |
| **Mixed methods research design** |
| Statements about the mixed methods research design: |
| **Integration of the quantitative and qualitative components** |
| Statements about the point/type of integration: |
| Statements showing evidence of integration: |
| **Added value from using mixed methods research** |
| Statements about the insights gained from integrating methods: |
| **Limitations of using mixed methods research** |
| Statements about limitations of one method associated with the presence of the other method: |
| **Statements specific to the value of mixed methods research for palliative care** |
| Statements about the ways in which mixed methods research is useful for palliative care: |
