# Supplementary material for: A Methodological Review of Mixed Methods Research in Palliative and End-of-Life Care (2014–2019)
Source: Int J Environ Res Public Health. 2020 May 29;17(11):3853. doi: 10.3390/ijerph17113853 (PMC7312170; doi:10.3390/ijerph17113853)
Supplement: Supplementary file 1 [file ijerph-17-03853-s001.zip › Supplementary Material/Supplementary_Material_2.docx]

**Supplementary material 2: Coding scheme**

| **Study topic and purpose** | |
| --- | --- |
| Study topic | Care planning, place of care, transition and documentation  Caring, situations and relationships  Existential and ethical issues  Experiences of illness, well-being, needs and environment  Organizational or professional development  Symptom assessment and management  Other topics |
| Study purpose | Assessment of palliative care needs  Evaluation of an intervention in, or program or service for palliative care  Investigation of a research topic in palliative care  Development and evaluation of an intervention in, or program or service for palliative care  Development and validation of a quantitative instrument for palliative care  Development of a tool or model for palliative care |

| **Mixed methods research component** | |
| --- | --- |
| **Description of mixed methods research** | |
| Description of the study as mixed methods research | Yes  No |
| Citation of key literature on mixed methods research | Yes  No |
| **Justification for using mixed methods research** | |
| Justification for using mixed methods research | Complementarity  Development to inform data collection  Development to inform sampling  Triangulation  Not reported / Not clear |
| **Mixed methods research question and design** | |
| Mixed methods research design | Convergent  Multistage  Exploratory sequential  Explanatory sequential  Not reported / Not clear |
| **Integration of the quantitative and qualitative components** | |
| Integration at the methods level | Connecting  Building  Merging  Not reported / Not clear |
| Integration at the reporting level | Narrative  Data transformation  Joint display  Not reported / Not clear |

| **Mixed methods reporting** | |
| --- | --- |
| **GRAMMS guidelines** | |
| 1. Describes the justification for using a mixed methods approach to the research question | Yes  Yes, but  No |
| 1. Describes the design in terms of the purpose, priority and sequence of methods | Yes  Yes, but  No |
| 1. Describes each method in terms of sampling, data collection and analysis | Yes  Yes, but  No |
| 1. Describes the integration of the quantitative and qualitative components | Yes  Yes, but  No |
| 1. Describes any limitation of one method associated with the presence of the other method | Yes  Yes, but  No |
| 1. Describes any insights gained from mixing or integrating methods | Yes  Yes, but  No |

**The Good Reporting of a Mixed Methods Study (GRAMMS) Criteria**

| **GRAMMS Criteria** | **Yes** | **Yes, but** | **No** |
| --- | --- | --- | --- |
| 1. Describes the justification for using a mixed methods approach to the research question | Provides an explicit justification for using mixed methods research. | Does not provide an explicit justification for using mixed methods research, but this justification can still be inferred. | Does not provide an explicit justification for using mixed methods research, and this justification cannot be inferred. |
| 1. Describes the design in terms of the purpose, priority and sequence of methods | Specifies the type of mixed methods research design used and describes both the priority and the timing of the quantitative and qualitative components. | Specifies the type of mixed methods research design used and describes either the priority or the timing of the quantitative and qualitative components. | Does not specify the type of mixed methods research design used, although it may describe either the priority or the timing of the methods used, or both. |
| 1. Describes each method in terms of sampling, data collection and analysis | Describes all (six) the elements of the quantitative and qualitative components (i.e., type of sampling, type of data sources, type of analyses). | Describes five or four elements of the quantitative and qualitative components (i.e., type of sampling, type of data sources, type of analyses). | Describes less than four elements of the quantitative and qualitative components (i.e., type of sampling, type of data sources, type of analyses). |
| 1. Describes the integration of the quantitative and qualitative components | Reports evidence of integration. It might also provide an explicit description of where and how integration has occurred, or else this information can be inferred. | Does not report evidence of integration, but it provides an explicit description of where and how integration has occurred. | Does not report evidence of integration and does not provide an explicit description of where and how integration has occurred. It either refers to the attempt of integrating the quantitative and qualitative components or uses keywords associated with integration, or both. |
| 1. Describes any limitation of one method associated with the presence of the other method | Provides an explicit description of the limitation. | Does not provide an explicit description of the limitation, but this limitation can still be inferred. | Does not provide an explicit description of the limitation and this limitation cannot be inferred. |
| 1. Describes any insights gained from mixing or integrating methods | Provides an explicit description of the insights gained from mixing or integrating methods. | Does not provide an explicit description of the insights, but these insights can still be inferred. | Does not provide an explicit description of the insights and these insights cannot be inferred. |
