# Supplementary material for: A Methodological Review of Mixed Methods Research in Palliative and End-of-Life Care (2014–2019)
Source: Int J Environ Res Public Health. 2020 May 29;17(11):3853. doi: 10.3390/ijerph17113853 (PMC7312170; doi:10.3390/ijerph17113853)
Supplement: Supplementary file 1 [file ijerph-17-03853-s001.zip › Supplementary Material/Supplementary_Material_3.docx]

**Supplementary material 3: Articles reporting mixed methods research included in the review**

[1-159]

1. Ankuda, C.K.; Kersting, K.; Guetterman, T.C.; Haefner, J.; Fonger, E.; Paletta, M.; Hopp, F. What matters most? A mixed methods study of critical aspects of a home-based palliative program. *Am J Hosp Palliat Care* **2018**, *35*, 236-243, doi:10.1177/1049909117691929.

2. Bergman, J.; Ballon-Landa, E.; Lorenz, K.A.; Saucedo, J.; Saigal, C.S.; Bennett, C.J.; Litwin, M.S. Community-Partnered Collaboration to Build an Integrated Palliative Care Clinic: The View From Urology. *Am J Hosp Palliat Care* **2016**, *33*, 164-170, doi:10.1177/1049909114555156.

3. Claxton-Oldfield, S.; Marrison-Shaw, H. Perceived Barriers and Enablers to Referrals to Community-Based Hospice Palliative Care Volunteer Programs in Canada. *Am J Hosp Palliat Care* **2014**, *31*, 836-844, doi:10.1177/1049909113504482.

4. Colclough, Y.Y.; Brown, G.M. Moving Toward Openness: Blackfeet Indians’ Perception Changes Regarding Talking About End of Life. *Am J Hosp Palliat Care* **2019**, *36*, 282-289, doi:10.1177/1049909118818255.

5. Dillon, E.; Chuang, J.; Gupta, A.; Tapper, S.; Lai, S.; Yu, P.; Ritchie, C.; Tai-Seale, M. Provider Perspectives on Advance Care Planning Documentation in the Electronic Health Record: The Experience of Primary Care Providers and Specialists Using Advance Health-Care Directives and Physician Orders for Life-Sustaining Treatment. *Am J Hosp Palliat Care* **2017**, *34*, 918-924, doi:10.1177/1049909117693578.

6. Hagiwara, Y.; Ross, J.; Lee, S.; Sanchez-Reilly, S. Tough Conversations: Development of a Curriculum for Medical Students to Lead Family Meetings. *Am J Hosp Palliat Care* **2017**, *34*, 907-911, doi:10.1177/1049909116669783.

7. Hernández-Marrero, P.; Pereira, S.M.; Carvalho, A.S. Ethical Decisions in Palliative Care: Interprofessional Relations as a Burnout Protective Factor? Results From a Mixed-Methods Multicenter Study in Portugal. *Am J Hosp Palliat Care* **2016**, *33*, 723-732, doi:10.1177/1049909115583486.

8. Kukulka, K.; Washington, K.T.; Govindarajan, R.; Mehr, D.R. Stakeholder Perspectives on the Biopsychosocial and Spiritual Realities of Living With ALS: Implications for Palliative Care Teams. *Am J Hosp Palliat Care* **2019**, *36*, 851-857, doi:10.1177/1049909119834493.

9. Levy, K.; Grant, P.C.; Depner, R.M.; Tenzek, K.E.; Pailler, M.E.; Beaupin, L.K.; Breier, J.M.; Byrwa, D.J. The Photographs of Meaning Program for Pediatric Palliative Caregivers: Feasibility of a Novel Meaning-Making Intervention. In *Am J Hosp Palliat Care*, 2019; Vol. 36, pp 557-563.

10. Lin, R.J.; Reid, M.C.; Liu, L.L.; Chused, A.E.; Evans, A.T. The Barriers to High-Quality Inpatient Pain Management: A Qualitative Study. *Am J Hosp Palliat Care* **2015**, *32*, 594-599, doi:10.1177/1049909114530491.

11. Lum, H.D.; Horney, C.; Koets, D.; Kutner, J.S.; Matlock, D.D. Availability of Heart Failure Medications in Hospice Care. *Am J Hosp Palliat Care* **2016**, *33*, 924-928, doi:10.1177/1049909115603689.

12. Meyer, D.; Schmidt, P.; Zernikow, B.; Wager, J. It’s All About Communication: A Mixed-Methods Approach to Collaboration Between Volunteers and Staff in Pediatric Palliative Care. *Am J Hosp Palliat Care* **2018**, *35*, 951-958, doi:10.1177/1049909117751419.

13. Nan, J.K.M.; Lau, B.H.P.; Szeto, M.M.L.; Lam, K.K.F.; Man, J.C.N.; Chan, C.L.W. Competence Enhancement Program of Expressive Arts in End-of-Life Care for Health and Social Care Professionals: A Mixed-Method Evaluation. *Am J Hosp Palliat Care* **2018**, *35*, 1207-1214, doi:10.1177/1049909118765410.

14. Nussbaum, S.E.; Oyola, S.; Egan, M.; Baron, A.; Wackman, S.; Williams, S.; Benson, J.; Limaye, S.; Levine, S. Incorporating Older Adults as “Trained Patients” to Teach Advance Care Planning to Third-Year Medical Students. *Am J Hosp Palliat Care* **2019**, *36*, 608-615, doi:10.1177/1049909119836394.

15. Parker, O.; Demiris, G.; Washington, K.; Kruse, R.L.; Petroski, G. Hospice Family Caregiver Involvement in Care Plan Meetings: A Mixed-Methods Randomized Controlled Trial. *Am J Hosp Palliat Care* **2017**, *34*, 849-859, doi:10.1177/1049909116661816.

16. Peng, C.S.; Baxter, K.; Lally, K.M. Music Intervention as a Tool in Improving Patient Experience in Palliative Care. *Am J Hosp Palliat Care* **2019**, *36*, 45-49, doi:10.1177/1049909118788643.

17. Rabow, M.W.; McGowan, M.; Small, R.; Keyssar, R.; Rugo, H.S. Advance Care Planning in Community: An Evaluation of a Pilot 2-Session, Nurse-Led Workshop. *Am J Hosp Palliat Care* **2019**, *36*, 143-146, doi:10.1177/1049909118797612.

18. Radha, K.; L, K.; Alsuwaigh, R.; Techna, M.; Shin, W.; Hui, L.; Manoharan, D. The influence of the family in conceptions of personhood in the palliative care setting in Singapore and its influence upon decision making. *Am J Hosp Palliat Care* **2014**, *31*, 645-654, doi:10.1177/1049909113500136.

19. Reese, D.J.; Beckwith, S.K. Organizational Barriers to Cultural Competence in Hospice. *Am J Hosp Palliat Care* **2015**, *32*, 685-694, doi:10.1177/1049909113520614.

20. Reese, D.J.; Buila, S.; Cox, S.; Davis, J.; Olsen, M.; Jurkowski, E. University–Community–Hospice Partnership to Address Organizational Barriers to Cultural Competence. *Am J Hosp Palliat Care* **2017**, *34*, 64-78, doi:10.1177/1049909115607295.

21. Tan, L.; Sim, L.K.; Ng, L.; Toh, H.J.; Low, J.A. Advance Care Planning: The Attitudes and Views of a Group of Catholic Nuns in Singapore. *Am J Hosp Palliat Care* **2017**, *34*, 26-33, doi:10.1177/1049909115615563.

22. Tanabe, K.; Sawada, K.; Shimada, M.; Kadoya, S.; Endo, N.; Ishiguro, K.; Takashima, R.; Amemiya, Y.; Fujikawa, Y.; Ikezaki, T., et al. Evaluation of A Novel Information-Sharing Instrument for Home-Based Palliative Care: A Feasibility Study. *Am J Hosp Palliat Care* **2015**, *32*, 611-619, doi:10.1177/1049909114533141.

23. Van, S.; L, J.; Green, M.J.; Reading, J.M.; Scott, A.M.; Chuang, C.H.; Levi, B.H. Can Playing an End-of-Life Conversation Game Motivate People to Engage in Advance Care Planning? *Am J Hosp Palliat Care* **2017**, *34*, 754-761, doi:10.1177/1049909116656353.

24. Van, S.; L, J.; Watson-Martin, E.; Bohr, T.A.; Levi, B.H.; Green, M.J. End-of-Life Conversation Game Increases Confidence for Having End-of-Life Conversations for Chaplains-in-Training. *Am J Hosp Palliat Care* **2018**, *35*, 592-600, doi:10.1177/1049909117723619.

25. Wagner, C.D.; Johns, S.; Brown, L.F.; Hanna, N.; Bigatti, S.M. Acceptability and Feasibility of a Meaning-Based Intervention for Patients With Advanced Cancer and Their Spouses: A Pilot Study. *Am J Hosp Palliat Care* **2016**, *33*, 546-554, doi:10.1177/1049909115575709.

26. Weisse, C.S.; Melekis, K.; Hutchins, B. Providing End-of-Life Care: Increased Empathy and Self-efficacy Among Student Caregivers in Residential Homes for the Dying. In *Am J Hosp Palliat Care*, 2018; Vol. 36, pp 538-545.

27. Afshar, K.; Feichtner, A.; Boyd, K.; Murray, S.; Jünger, S.; Wiese, B.; Schneider, N.; Müller-Mundt, G. Systematic development and adjustment of the German version of the Supportive and Palliative Care Indicators Tool (SPICT-DE). *BMC Palliat Care* **2018**, *17*, doi:10.1186/s12904-018-0283-7.

28. Ateş, G.; Ebenau, A.F.; Busa, C.; Csikos, Á.; Hasselaar, J.; Jaspers, B.; Menten, J.; Payne, S.; Van, B.; Varey, S., et al. "never at ease" - Family carers within integrated palliative care: A multinational, mixed method study. *BMC Palliat Care* **2018**, *17*, doi:10.1186/s12904-018-0291-7.

29. Beaussant, Y.; Mathieu-Nicot, F.; Pazart, L.; Tournigand, C.; Daneault, S.; Cretin, E.; Godard-Marceau, A.; Chassagne, A.; Trimaille, H.; Bouleuc, C., et al. Is shared decision-making vanishing at the end-of-life? A descriptive and qualitative study of advanced cancer patient's involvement in specific therapies decision-making Cancer palliative care. *BMC Palliat Care* **2015**, *14*, doi:10.1186/s12904-015-0057-4.

30. Bergenholtz, H.; Hølge-Hazelton, B.; Jarlbaek, L. Organization and evaluation of generalist palliative care in a Danish hospital. *BMC Palliat Care* **2015**, *14*, doi:10.1186/s12904-015-0022-2.

31. Bush, S.H.; Grassau, P.A.; Yarmo, M.N.; Zhang, T.; Zinkie, S.J.; Pereira, J.L. The Richmond Agitation-Sedation Scale modified for palliative care inpatients (RASS-PAL): A pilot study exploring validity and feasibility in clinical practice. *BMC Palliat Care* **2014**, *13*, doi:10.1186/1472-684X-13-17.

32. Costantini, M.; Rabitti, E.; Beccaro, M.; Fusco, F.; Peruselli, C.; La, C.; Valle, A.; Suriani, C.; Berardi, M.A.; Valenti, D., et al. Validity, reliability and responsiveness to change of the Italian palliative care outcome scale: A multicenter study of advanced cancer patients Cancer palliative care. *BMC Palliat Care* **2016**, *15*, doi:10.1186/s12904-016-0095-6.

33. De, K.-V.; M, C.; Pasman, H.R.W.; Schweitzer, B.P.; Francke, A.L.; Onwuteaka-Philipsen, B.D.; Deliens, L. Burden for family carers at the end of life; A mixed-method study of the perspectives of family carers and GPs. *BMC Palliat Care* **2014**, *13*, doi:10.1186/1472-684X-13-16.

34. Downing, J.; Batuli, M.; Kivumbi, G.; Kabahweza, J.; Grant, L.; Murray, S.A.; Namukwaya, E.; Leng, M. A palliative care link nurse programme in Mulago Hospital, Uganda: An evaluation using mixed methods. *BMC Palliat Care* **2016**, *15*, doi:10.1186/s12904-016-0115-6.

35. Farrington, C.J.T. Blended e-learning and end of life care in nursing homes: A small-scale mixed-methods case study. *BMC Palliat Care* **2014**, *13*, doi:10.1186/1472-684X-13-31.

36. Fleming, R.; Kelly, F.; Stillfried, G. 'I want to feel at home': Establishing what aspects of environmental design are important to people with dementia nearing the end of life Palliative care in other conditions. *BMC Palliat Care* **2015**, *14*, doi:10.1186/s12904-015-0026-y.

37. Friedel, M.; Brichard, B.; Fonteyne, C.; Renard, M.; Misson, J.P.; Vandecruys, E.; Tonon, C.; Verfaillie, F.; Hendrijckx, G.; Andersson, N., et al. Building Bridges, Paediatric Palliative Care in Belgium: A secondary data analysis of annual paediatric liaison team reports from 2010 to 2014. *BMC Palliat Care* **2018**, *17*, doi:10.1186/s12904-018-0324-2.

38. Henriksen, K.M.E.; Heller, N.; Finucane, A.M.; Oxenham, D. Is the patient satisfaction questionnaire an acceptable tool for use in a hospice inpatient setting? A pilot study. *BMC Palliat Care* **2014**, *13*, doi:10.1186/1472-684X-13-27.

39. Iliffe, S.; Davies, N.; Manthorpe, J.; Crome, P.; Ahmedzai, S.H.; Vernooij-Dassen, M.; Engels, Y. Improving palliative care in selected settings in England using quality indicators: A realist evaluation. *BMC Palliat Care* **2016**, *15*, doi:10.1186/s12904-016-0144-1.

40. Johnston, B.; Patterson, A.; Bird, L.; Wilson, E.; Almack, K.; Mathews, G.; Seymour, J. Impact of the Macmillan specialist Care at Home service: A mixed methods evaluation across six sites. *BMC Palliat Care* **2018**, *17*, doi:10.1186/s12904-018-0281-9.

41. Johnston, B.; Pringle, J.; Gaffney, M.; Narayanasamy, M.; McGuire, M.; Buchanan, D. The dignified approach to care: A pilot study using the patient dignity question as an intervention to enhance dignity and person-centred care for people with palliative care needs in the acute hospital setting Psychosocial. *BMC Palliat Care* **2015**, *14*, doi:10.1186/s12904-015-0013-3.

42. Knighting, K.; O'Brien, M.R.; Roe, B.; Gandy, R.; Lloyd-Williams, M.; Nolan, M.; Jack, B.A. Development of the Carers' Alert Thermometer (CAT) to identify family carers struggling with caring for someone dying at home: A mixed method consensus study. *BMC Palliat Care* **2015**, *14*, doi:10.1186/s12904-015-0010-6.

43. Mai, S.S.; Goebel, S.; Jentschke, E.; Van, O.; Renner, K.H.; Weber, M. Feasibility, acceptability and adaption of dignity therapy: A mixed methods study achieving 360° feedback. *BMC Palliat Care* **2018**, *17*, doi:10.1186/s12904-018-0326-0.

44. Martins, P.; Araújo, J.; Hernández-Marrero, P. Towards a public health approach for palliative care: An action-research study focused on engaging a local community and educating teenagers. *BMC Palliat Care* **2018**, *17*, doi:10.1186/s12904-018-0344-y.

45. Massey, K.; Barnes, M.J.; Villines, D.; Goldstein, J.D.; Pierson, A.L.H.; Scherer, C.; Laan, B.V.; Summerfelt, W.T. What do i do? Developing a taxonomy of chaplaincy activities and interventions for spiritual care in intensive care unit palliative care Palliative Care. *BMC Palliat Care* **2015**, *14*, doi:10.1186/s12904-015-0008-0.

46. Mayrhofer, A.; Goodman, C.; Smeeton, N.; Handley, M.; Amador, S.; Davies, S. The feasibility of a train-the-trainer approach to end of life care training in care homes: An evaluation. *BMC Palliat Care* **2016**, *15*, doi:10.1186/s12904-016-0081-z.

47. Michael, N.; O'Callaghan, C.; Baird, A.; Gough, K.; Krishnasamy, M.; Hiscock, N.; Clayton, J. A mixed method feasibility study of a patient- and family-centred advance care planning intervention for cancer patients. *BMC Palliat Care* **2015**, *14*, doi:10.1186/s12904-015-0023-1.

48. Orellana-Rios, C.L.; Radbruch, L.; Kern, M.; Regel, Y.U.; Anton, A.; Sinclair, S.; Schmidt, S. Mindfulness and compassion-oriented practices at work reduce distress and enhance self-care of palliative care teams: A mixed-method evaluation of an "on the job" program. *BMC Palliat Care* **2017**, *17*, doi:10.1186/s12904-017-0219-7.

49. Payne, S.; Hughes, S.; Wilkinson, J.; Hasselaar, J.; Preston, N. Recommendations on priorities for integrated palliative care: Transparent expert consultation with international leaders for the InSuP-C project. *BMC Palliat Care* **2019**, *18*, doi:10.1186/s12904-019-0418-5.

50. Pesut, B.; Duggleby, W.; Warner, G.; Fassbender, K.; Antifeau, E.; Hooper, B.; Greig, M.; Sullivan, K. Volunteer navigation partnerships: Piloting a compassionate community approach to early palliative care. *BMC Palliat Care* **2017**, *17*, doi:10.1186/s12904-017-0210-3.

51. Pesut, B.; Hooper, B.; Jacobsen, M.; Nielsen, B.; Falk, M.; O‘Connor, B.P. Nurse-led navigation to provide early palliative care in rural areas: a pilot study. *BMC Palliat Care* **2017**, *16*, doi:10.1186/s12904-017-0211-2.

52. Preissler, P.; Kordovan, S.; Ullrich, A.; Bokemeyer, C.; Oechsle, K. Favored subjects and psychosocial needs in music therapy in terminally ill cancer patients: A content analysis. *BMC Palliat Care* **2016**, *15*, doi:10.1186/s12904-016-0122-7.

53. Stöckle, H.S.; Haarmann-Doetkotte, S.; Bausewein, C.; Fegg, M.J. The feasibility and acceptability of short-term, individual existential behavioural therapy for informal caregivers of patients recruited in a specialist palliative care unit. *BMC Palliat Care* **2016**, *15*, 1-10, doi:10.1186/s12904-016-0160-1.

54. Taubert, M.; Norris, J.; Edwards, S.; Snow, V.; Finlay, I.G. Talk CPR - A technology project to improve communication in do not attempt cardiopulmonary resuscitation decisions in palliative illness 11 Medical and Health Sciences 1117 Public Health and Health Services. *BMC Palliat Care* **2018**, *17*, doi:10.1186/s12904-018-0370-9.

55. Thompson, G.N.; McClement, S.E.; Labun, N.; Klaasen, K. Developing and testing a nursing home end -of -life care chart audit tool. *BMC Palliat Care* **2018**, *17*, doi:10.1186/s12904-018-0301-9.

56. Trarieux-Signol, S.; Moreau, S.; Gourin, M.P.; Penot, A.; Edoux De, L.; Preux, P.M.; Bordessoule, D. Factors associated with the designation of a health care proxy and writing advance directives for patients suffering from haematological malignancies. *BMC Palliat Care* **2014**, *13*, doi:10.1186/1472-684X-13-57.

57. Verhofstede, R.; Smets, T.; Cohen, J.; Costantini, M.; Van Den, N.; Deliens, L. Implementing the care programme for the last days of life in an acute geriatric hospital ward: A phase 2 mixed method study. *BMC Palliat Care* **2016**, *15*, doi:10.1186/s12904-016-0102-y.

58. Widger, K.; Tourangeau, A.E.; Steele, R.; Streiner, D.L. Initial development and psychometric testing of an instrument to measure the quality of children's end-of-life care. *BMC Palliat Care* **2015**, *14*, doi:10.1186/1472-684X-14-1.

59. Addington-Hall, J.; Hunt, K.; Rowsell, A.; Heal, R.; Hansford, P.; Monroe, B.; Sykes, N. Development and initial validation of a new outcome measure for hospice and palliative care: The St Christopher's Index of Patient Priorities (SKIPP). *BMJ Support Palliat Care* **2014**, *4*, 175-181, doi:10.1136/bmjspcare-2012-000352.

60. Buck, J.; Webb, L.; Moth, L.; Morgan, L.; Barclay, S. Persistent inequalities in Hospice at Home provision. In *BMJ Support Palliat Care*, 2018; 10.1136/bmjspcare-2017-001367.

61. Cheong, K.; Fisher, P.; Goh, J.; Ng, L.; Koh, H.M.; Yap, P. Advance care planning in people with early cognitive impairment. *BMJ Support Palliat Care* **2015**, *5*, 63-69, doi:10.1136/bmjspcare-2014-000648.

62. Gracey, J.H.; Watson, M.; Payne, C.; Rankin, J.; Dunwoody, L. Translation research: 'Back on Track', a multiprofessional rehabilitation service for cancer-related fatigue. *BMJ Support Palliat Care* **2016**, *6*, 94-96, doi:10.1136/bmjspcare-2014-000692.

63. Harley, C.; Pini, S.; Kenyon, L.; Daffu-O'Reilly, A.; Velikova, G. Evaluating the experiences and support needs of people living with chronic cancer: Development and initial validation of the Chronic Cancer Experiences Questionnaire (CCEQ). *BMJ Support Palliat Care* **2019**, *9*, e15, doi:10.1136/bmjspcare-2015-001032.

64. Highet, G.; Crawford, D.; Murray, S.A.; Boyd, K. Development and evaluation of the Supportive and Palliative Care Indicators Tool (SPICT): A mixed-methods study. *BMJ Support Palliat Care* **2014**, *4*, 285-290, doi:10.1136/bmjspcare-2013-000488.

65. Hunt, K.J.; Richardson, A.; Darlington, A.S.E.; Addington-Hall, J.M. Developing the methods and questionnaire (VOICES-SF) for a national retrospective mortality follow-back survey of palliative and end-of-life care in England. In *BMJ Support Palliat Care*, 2019; Vol. 9.

66. Ingold, K.; Hicks, F. Using a public health approach to improve end-of-life care: Results and discussion of a health needs assessment undertaken in a large city in northern England. *BMJ Support Palliat Care* **2015**, *5*, 200-202, doi:10.1136/bmjspcare-2014-000662.

67. Kane, P.M.; Daveson, B.A.; Ryan, K.; Ellis-Smith, C.I.; Mahon, N.G.; McAdam, B.; McQuilllan, R.; Tracey, C.; Howley, C.; O'Gara, G., et al. Feasibility and acceptability of a patient-reported outcome intervention in chronic heart failure. *BMJ Support Palliat Care* **2017**, *7*, 470-479, doi:10.1136/bmjspcare-2017-001355.

68. Mayland, C.R.; Lees, C.; Germain, A.; Jack, B.A.; Cox, T.F.; Mason, S.R.; West, A.; Ellershaw, J.E. Caring for those who die at home: The use and validation of 'Care Of the Dying Evaluation' (CODE) with bereaved relatives. *BMJ Support Palliat Care* **2014**, *4*, 167-174, doi:10.1136/bmjspcare-2013-000596.

69. Sandsund, C.; Towers, R.; Thomas, K.; Tigue, R.; Lalji, A.; Fernandes, A.; Doyle, N.; Jordan, J.; Gage, H.; Shaw, C. Holistic needs assessment and care plans for women with gynaecological cancer: Do they improve cancer-specific health-related quality of life? A randomised controlled trial using mixed methods. In *BMJ Support Palliat Care*, 2017; 10.1136/bmjspcare-2016-001207.

70. Shaw, K.L.; Brook, L.; Mpundu-Kaambwa, C.; Harris, N.; Lapwood, S.; Randall, D. The spectrum of children's palliative care needs: A classification framework for children with life-limiting or life-threatening conditions. *BMJ Support Palliat Care* **2015**, *5*, 249-258, doi:10.1136/bmjspcare-2012-000407.

71. Sweeney, C.; Lynch, G.; Khashan, A.; Maher, B.; Murphy, M.; O'Brien, T. The impact of a medical undergraduate student-selected module in palliative care. *BMJ Support Palliat Care* **2014**, *4*, 92-97, doi:10.1136/bmjspcare-2012-000283.

72. Taylor, P.; Johnson, M.J.; Dowding, D.W. Clinical decision-making at the end of life: A mixed-methods study. In *BMJ Support Palliat Care*, 2018; 10.1136/bmjspcare-2018-001535.

73. Tsai, G.; Taylor, D.H. Advance care planning in Medicare: An early look at the impact of new reimbursement on billing and clinical practice. *BMJ Support Palliat Care* **2018**, *8*, 49-52, doi:10.1136/bmjspcare-2016-001181.

74. White, C.; Sproule, J.; Brogan, P.; Watson, M. Patient healthcare passports in community specialist palliative care: A mixed methods study. In *BMJ Support Palliat Care*, 2018; 10.1136/bmjspcare-2018-001589.

75. Zweers, D.; de Graaf, E.; Teunissen, S. Suitable support for anxious hospice patients: What do nurses 'know', 'do' and 'need'? An explanatory mixed method study. In *BMJ Support Palliat Care*, 2017; 10.1136/bmjspcare-2016-001187.

76. Dobrina, R.; Tenze, M.; Palese, A. Transforming End-of-Life Care by Implementing a Patient-Centered Care Model: Findings from an Action Research Project. *J Hosp Palliat Nurs* **2018**, *20*, 531-541, doi:10.1097/NJH.0000000000000468.

77. Jeffers, S. Integration of a Hospice Clinical Experience: Nursing Students' Perceptions. *J Hosp Palliat Nurs* **2018**, *20*, 266-271, doi:10.1097/NJH.0000000000000437.

78. Long, M.B.; Bekelman, D.B.; Make, B. Improving quality of life in chronic obstructive pulmonary disease by integrating palliative approaches to dyspnea, anxiety, and depression. *J Hosp Palliat Nurs* **2014**, *16*, 514-520, doi:10.1097/NJH.0000000000000111.

79. Lowey, S.E.; Liebel, D.V. Factors that influence care transitions of end-stage heart failure patients to palliative home care. *J Hosp Palliat Nurs* **2016**, *18*, 572-578, doi:10.1097/NJH.0000000000000296.

80. Mager, D.R.; Lange, J.W. The elder project: Evaluating end-of-life knowledge among health care providers. *J Hosp Palliat Nurs* **2016**, *18*, 22-28, doi:10.1097/NJH.0000000000000199.

81. O'Mallon, M.O. Bereavement: Exploring perceived social support and family relationships of selected family caregivers. *J Hosp Palliat Nurs* **2014**, *16*, 304-311, doi:10.1097/NJH.0000000000000071.

82. Tan, L.; Gan, G.; Hum, A.; Lee, A. A Stepwise, Mixed-Method Study Approach to Identify the Barriers to Dysphagia Care in Hospice Care Nurses. *J Hosp Palliat Nurs* **2018**, *20*, 88-94, doi:10.1097/NJH.0000000000000404.

83. Whitehead, P.B.; Ramalingam, N.; Carter, K.F.; Katz, K.; Harden, S. Nurse practitioners' perspectives on the patient preferences about serious illness instrument. *J Hosp Palliat Nurs* **2016**, *18*, 332-341, doi:10.1097/NJH.0000000000000256.

84. Brand, A.H.; Harrison, A.; Kumar, K. "It was definitely very different": An evaluation of palliative care teaching to medical students using a mixed methods approach. *J Palliat Care* **2015**, *31*, 21-28, doi:10.1177/082585971503100104.

85. Rice, J.; Hunter, L.; Hsu, A.T.; Donskov, M.; Luciani, T.; Toal-Sullivan, D.; Welch, V.; Tanuseputro, P. Using the “surprise question” in nursing homes: A prospective mixed-methods study. *J Palliat Care* **2018**, *33*, 9-18, doi:10.1177/0825859717745728.

86. Sarti, A.J.; Bourbonnais, F.F.; Landriault, A.; Sutherland, S.; Cardinal, P. An interhospital, interdisciplinary needs assessment of palliative care in a community critical care context. *J Palliat Care* **2015**, *31*, 234-242, doi:10.1177/082585971503100405.

87. Bekelman, D.B.; Hooker, S.; Nowels, C.T.; Main, D.S.; Meek, P.; McBryde, C.; Hattler, B.; Lorenz, K.A.; Heidenreich, P.A. Feasibility and acceptability of a collaborative care intervention to improve symptoms and quality of life in chronic heart failure: Mixed methods pilot trial. *J Palliat Med* **2014**, *17*, 145-151, doi:10.1089/jpm.2013.0143.

88. Bekelman, D.B.; Johnson-Koenke, R.; Bowles, D.W.; Fischer, S.M. Improving early palliative care with a scalable, stepped peer navigator and social work intervention: A single-arm clinical trial. *J Palliat Med* **2018**, *21*, 1011-1016, doi:10.1089/jpm.2017.0424.

89. Ceronsky, L.; Johnson, L.G.; Weng, K. Quality Measures for Community-Based, Rural Palliative Care Programs in Minnesota: A Pilot Study. *J Palliat Med* **2015**, *18*, 618-624, doi:10.1089/jpm.2014.0435.

90. Coats, H.; Paganelli, T.; Starks, H.; Lindhorst, T.; Starks, A.; Mauksch, L.; Doorenbos, A. A Community Needs Assessment for the Development of an Interprofessional Palliative Care Training Curriculum. *J Palliat Med* **2017**, *20*, 235-240, doi:10.1089/jpm.2016.0321.

91. Cooper, Z.; Corso, K.; Bernacki, R.; Bader, A.; Gawande, A.; Block, S. Conversations about treatment preferences before high-risk surgery: A pilot study in the preoperative testing center. *J Palliat Med* **2014**, *17*, 701-707, doi:10.1089/jpm.2013.0311.

92. Dionne-Odom, J.N.; Kono, A.; Frost, J.; Jackson, L.; Ellis, D.; Ahmed, A.; Azuero, A.; Bakitas, M. Translating and testing the ENABLE: CHF-PC concurrent palliative care model for older adults with heart failure and their family caregivers. *J Palliat Med* **2014**, *17*, 995-1004, doi:10.1089/jpm.2013.0680.

93. Harrison, K.L.; Boyden, J.Y.; Kalish, V.B.; Muir, J.C.; Richardson, S.; Connor, S.R. A Hospice Rotation for Military Medical Residents: A Mixed Methods, Multi-Perspective Program Evaluation. *J Palliat Med* **2016**, *19*, 542-548, doi:10.1089/jpm.2015.0339.

94. Hickman, S.E.; Nelson, C.A.; Smith-Howell, E.; Hammes, B.J. Use of the physician orders for life-sustaining treatment program for patients being discharged from the hospital to the nursing facility. *J Palliat Med* **2014**, *17*, 43-49, doi:10.1089/jpm.2013.0097.

95. Hobler, M.R.; Engelberg, R.A.; Curtis, J.R.; Ramos, K.J.; Zander, M.I.; Howard, S.S.; Goss, C.H.; Aitken, M.L. Exploring Opportunities for Primary Outpatient Palliative Care for Adults with Cystic Fibrosis: A Mixed-Methods Study of Patients' Needs. *J Palliat Med* **2018**, *21*, 513-521, doi:10.1089/jpm.2017.0259.

96. Huang, C.H.S.; Crowther, M.; Allen, R.S.; Decoster, J.; Kim, G.; Azuero, C.; Ang, X.; Kvale, E. A pilot feasibility intervention to increase advance care planning among African Americans in the deep south. *J Palliat Med* **2016**, *19*, 164-173, doi:10.1089/jpm.2015.0334.

97. Izumi, S.S.; Basin, B.; Presley, M.; McCalmont, J.; Furuno, J.P.; Noble, B.; Baggs, J.G.; Curtis, J.R. Feasibility and Acceptability of Nurse-Led Primary Palliative Care for Older Adults with Chronic Conditions: A Pilot Study. *J Palliat Med* **2018**, *21*, 1114-1121, doi:10.1089/jpm.2017.0666.

98. Lewin, W.H.; Cheung, W.; Horvath, A.N.; Haberman, S.; Patel, A.; Sullivan, D. Supportive Cardiology: Moving Palliative Care Upstream for Patients Living with Advanced Heart Failure. *J Palliat Med* **2017**, *20*, 1112-1119, doi:10.1089/jpm.2016.0317.

99. Low, D.; Merkel, E.C.; Menon, M.; Loggers, E.; Ddungu, H.; Leng, M.; Namukwaya, E.; Casper, C. End-of-Life Palliative Care Practices and Referrals in Uganda. *J Palliat Med* **2018**, *21*, 328-334, doi:10.1089/jpm.2017.0257.

100. Monterosso, L.; Ross-Adjie, G.M.; Rogers, I.R.; Shearer, F.M.; Rogers, J.R. How Well Do We Understand Health Care Professionals' Perceptions and Needs in the Provision of Palliative Care? A Mixed Methods Study. *J Palliat Med* **2016**, *19*, 720-727, doi:10.1089/jpm.2015.0421.

101. Mooney-Doyle, K.; Deatrick, J.A.; Ulrich, C.M.; Meghani, S.H.; Feudtner, C. Parenting in Childhood Life-Threatening Illness: A Mixed-Methods Study. *J Palliat Med* **2018**, *21*, 208-215, doi:10.1089/jpm.2017.0054.

102. Myers, J.; Krueger, P.; Webster, F.; Downar, J.; Herx, L.; Jeney, C.; Oneschuk, D.; Schroder, C.; Sirianni, G.; Seccareccia, D., et al. Development and validation of a set of palliative medicine entrustable professional activities: Findings from a mixed methods study. *J Palliat Med* **2015**, *18*, 682-690, doi:10.1089/jpm.2014.0392.

103. Nedjat-Haiem, F.R.; Carrion, I.V.; Gonzalez, K.; Quintana, A.; Ell, K.; O'Connell, M.; Thompson, B.; Mishra, S.I. Implementing an Advance Care Planning Intervention in Community Settings with Older Latinos: A Feasibility Study. *J Palliat Med* **2017**, *20*, 984-993, doi:10.1089/jpm.2016.0504.

104. O'Connor, N.R.; Moyer, M.E.; Kirkpatrick, J.N. Scripted Nurse Visits: A Resource-Efficient Palliative Care Model for Ventricular Assist Devices. *J Palliat Med* **2016**, *19*, 1312-1315, doi:10.1089/jpm.2016.0065.

105. Rhee, J.Y.; Garralda, E.; Namisango, E.; Luyirika, E.; De, L.; Powell, R.A.; Centeno, C. Developing Macroindicators of Palliative Care Development in Africa: A Process with In-Country and International Experts. *J Palliat Med* **2018**, *21*, 342-353, doi:10.1089/jpm.2017.0207.

106. Sanders, J.J.; Chow, V.; Enzinger, A.C.; Lam, T.C.; Smith, P.T.; Quiñones, R.; Baccari, A.; Philbrick, S.; White-Hammond, G.; Peteet, J., et al. Seeking and Accepting: U.S. Clergy Theological and Moral Perspectives Informing Decision Making at the End of Life. *J Palliat Med* **2017**, *20*, 1059-1067, doi:10.1089/jpm.2016.0545.

107. Schenker, Y.; Bahary, N.; Claxton, R.; Childers, J.; Chu, E.; Kavalieratos, D.; King, L.; Lembersky, B.; Tiver, G.; Arnold, R.M. A Pilot Trial of Early Specialty Palliative Care for Patients with Advanced Pancreatic Cancer: Challenges Encountered and Lessons Learned. *J Palliat Med* **2018**, *21*, 28-36, doi:10.1089/jpm.2017.0113.

108. Szekendi, M.K.; Vaughn, J.; Lal, A.; Ouchi, K.; Williams, M.V. The Prevalence of Inpatients at 33 U.S. Hospitals Appropriate for and Receiving Referral to Palliative Care. *J Palliat Med* **2016**, *19*, 360-372, doi:10.1089/jpm.2015.0236.

109. Van, S.; L, J.; Reading, J.M.; Scott, A.M.; Green, M.J.; Levi, B.H. Conversation Game Effectively Engages Groups of Individuals in Discussions about Death and Dying. *J Palliat Med* **2016**, *19*, 661-667, doi:10.1089/jpm.2015.0390.

110. Buzgova, R.; Kozakova, R.; Sikorova, L.; Zelenikova, R.; Jarosova, D. Development and psychometric evaluation of patient needs assessment in palliative care (PNAP) instrument. *Palliat Support Care* **2016**, *14*, 129-137, doi:10.1017/S1478951515000061.

111. Camacho, A.A.; Garland, S.N.; Martopullo, C.; Pelletier, G. Positive and negative meanings are simultaneously ascribed to colorectal cancer: Relationship to quality of life and psychosocial adjustment. *Palliat Support Care* **2014**, *12*, 277-286, doi:10.1017/S1478951513000151.

112. Carlsson, M.E. A separate structured conversation with relatives of patients enrolled for advanced palliative home care: A care development project. *Palliat Support Care* **2014**, *12*, 107-115, doi:10.1017/S1478951512001022.

113. Cohen, M.M.; Wellisch, D.K.; Ormseth, S.R.; Yarema, V.G. The father-daughter relationship in the wake of maternal death from breast cancer. *Palliat Support Care* **2018**, *16*, 741-748, doi:10.1017/S1478951517000906.

114. D'Angelo, D.; Mastroianni, C.; Artico, M.; Biagioli, V.; Latina, R.; Guarda, M.; Piredda, M.; De, M.; M, G. Validity and reliability of the Palliative Care Transition Measure for Caregivers (PCTM-C). *Palliat Support Care* **2019**, *17*, 202-207, doi:10.1017/S1478951517001225.

115. Egan, R.; Macleod, R.; Jaye, C.; McGee, R.; Baxter, J.; Herbison, P.; Wood, S. Spiritual beliefs, practices, and needs at the end of life: Results from a New Zealand national hospice study. *Palliat Support Care* **2017**, *15*, 223-230, doi:10.1017/S147895151600064X.

116. Green, A.; Jerzmanowska, N.; Thristiawati, S.; Green, M.; Lobb, E.A. Culturally and linguistically diverse palliative care patients' journeys at the end-of-life. *Palliat Support Care* **2019**, *17*, 227-233, doi:10.1017/S1478951518000147.

117. Hudson, P.; Collins, A.; Bostanci, A.; Willenberg, L.; Stephanov, N.; Phillip, J. Toward a systematic approach to assessment and care planning in palliative care: A practical review of clinical tools. *Palliat Support Care* **2016**, *14*, 161-173, doi:10.1017/S1478951515000565.

118. Hudson, P.; Hall, C.; Boughey, A.; Roulston, A. Bereavement support standards and bereavement care pathway for quality palliative care. *Palliat Support Care* **2018**, *16*, 375-387, doi:10.1017/S1478951517000451.

119. Hudson, P.; Street, A.; Graham, S.; Aranda, S.; O'Connor, M.; Thomas, K.; Jackson, K.; Spruyt, O.; Ugalde, A.; Philip, J. Establishment and preliminary outcomes of a palliative care research network. *Palliat Support Care* **2014**, *14*, 52-59, doi:10.1017/S1478951515000723.

120. Lambie, D.; Egan, R.; Walker, S.; MacLeod, R. How spirituality is understood and taught in New Zealand medical schools. *Palliat Support Care* **2015**, *13*, 53-58, doi:10.1017/S147895151300062X.

121. Lind, S.; Sandberg, J.; Brytting, T.; Fürst, C.J.; Wallin, L. Implementation of the integrated palliative care outcome scale in acute care settings-a feasibility study. *Palliat Support Care* **2018**, *16*, 698-705, doi:10.1017/S1478951517001158.

122. Lind, S.; Wallin, L.; Fürst, C.J.; Beck, I. The integrated palliative care outcome scale for patients with palliative care needs: Factors related to and experiences of the use in acute care settings. *Palliat Support Care* **2019**, *17*, 561-568, doi:10.1017/S1478951518001104.

123. Olivier-D'Avignon, M.; Dumont, S.; Valois, P.; Cohen, S.R. The needs of siblings of children with a life-threatening illness, part 1: Conceptualization and development of a measure. *Palliat Support Care* **2017**, *15*, 644-664, doi:10.1017/S1478951516001127.

124. Philip, J.; Crawford, G.; Brand, C.; Gold, M.; Miller, B.; Hudson, P.; Smallwood, N.; Lau, R.; Sundararajan, V. A conceptual model: Redesigning how we provide palliative care for patients with chronic obstructive pulmonary disease. *Palliat Support Care* **2018**, *16*, 452-460, doi:10.1017/S147895151700044X.

125. Sand, L.; Olsson, M.; Strang, P. Supporting in an existential crisis: A mixed-methods evaluation of a training model in palliative care. *Palliat Support Care* **2018**, *16*, 470-478, doi:10.1017/S1478951517000633.

126. Steinhauser, K.E.; Olsen, A.; Johnson, K.S.; Sanders, L.L.; Olsen, M.; Ammarell, N.; Grossoehme, D. The feasibility and acceptability of a chaplain-led intervention for caregivers of seriously ill patients: A Caregiver Outlook pilot study. *Palliat Support Care* **2016**, *14*, 456-467, doi:10.1017/S1478951515001248.

127. Wittenberg, E.; Kravits, K.; Goldsmith, J.; Ferrell, B.; Fujinami, R. Validation of a model of family caregiver communication types and related caregiver outcomes. *Palliat Support Care* **2017**, *15*, 3-11, doi:10.1017/S1478951516000109.

128. Wittenberg-Lyles, E.; Washington, K.; Oliver, D.P.; Shaunfield, S.; Gage, L.A.; Mooney, M.; Lewis, A. It is the 'starting over' part that is so hard: Using an online group to support hospice bereavement. *Palliat Support Care* **2015**, *13*, 351-357, doi:10.1017/S1478951513001235.

129. Wu, L.M.; Kuprian, N.; Herbert, K.; Amidi, A.; Austin, J.; Valdimarsdottir, H.; Rini, C. A mixed methods analysis of perceived cognitive impairment in hematopoietic stem cell transplant survivors. In *Palliat Support Care*, 2018; 10.1017/S1478951518000664pp 1-7.

130. Bainbridge, D.; Brazil, K.; Ploeg, J.; Krueger, P.; Taniguchi, A. Measuring healthcare integration: Operationalization of a framework for a systems evaluation of palliative care structures, processes, and outcomes. *Palliat Med* **2016**, *30*, 567-579, doi:10.1177/0269216315619862.

131. Bergenholtz, H.; Jarlbaek, L.; Hølge-Hazelton, B. Generalist palliative care in hospital - Cultural and organisational interactions. Results of a mixed-methods study. *Palliat Med* **2016**, *30*, 558-566, doi:10.1177/0269216315619861.

132. Boss, R.D.; Hutton, N.; Griffin, P.L.; Wieczorek, B.H.; Donohue, P.K. Novel legislation for pediatric advance directives: Surveys and focus groups capture parent and clinician perspectives. *Palliat Med* **2015**, *29*, 346-353, doi:10.1177/0269216315571020.

133. Bristowe, K.; Carey, I.; Hopper, A.; Shouls, S.; Prentice, W.; Caulkin, R.; Higginson, I.J.; Koffman, J. Patient and carer experiences of clinical uncertainty and deterioration, in the face of limited reversibility: A comparative observational study of the AMBER care bundle. *Palliat Med* **2015**, *29*, 797-807, doi:10.1177/0269216315578990.

134. Bristowe, K.; Shepherd, K.; Bryan, L.; Brown, H.; Carey, I.; Matthews, B.; O'Donoghue, D.; Vinen, K.; Murtagh, F.E. The development and piloting of the renal specific advanced communication training (react) programme to improve advance care planning for renal patients. *Palliat Med* **2014**, *28*, 360-366, doi:10.1177/0269216313510342.

135. Costantini, M.; Apolone, G.; Tanzi, S.; Falco, F.; Rondini, E.; Guberti, M.; Fanello, S.; Cavuto, S.; Savoldi, L.; Piro, R., et al. Is early integration of palliative care feasible and acceptable for advanced respiratory and gastrointestinal cancer patients? A phase 2 mixed-methods study. *Palliat Med* **2018**, *32*, 46-58, doi:10.1177/0269216317731571.

136. Cowey, E.; Smith, L.N.; Stott, D.J.; McAlpine, C.H.; Mead, G.E.; Barber, M.; Walters, M. Impact of a clinical pathway on end-of-life care following stroke: A mixed methods study. *Palliat Med* **2015**, *29*, 249-259, doi:10.1177/0269216314551378.

137. Crocker, J.C.; Beecham, E.; Kelly, P.; Dinsdale, A.P.; Hemsley, J.; Jones, L.; Bluebond-Langner, M. Inviting parents to take part in paediatric palliative care research: A mixed-methods examination of selection bias. *Palliat Med* **2015**, *29*, 231-240, doi:10.1177/0269216314560803.

138. Dalkin, S.; Lhussier, M.; Jones, D.; Phillipson, P.; Cunningham, W. Open communication strategies between a triad of ‘experts’ facilitates death in usual place of residence: A realist evaluation. *Palliat Med* **2018**, *32*, 980-989, doi:10.1177/0269216318757132.

139. Dalkin, S.M.; Lhussier, M.; Philipson, P.; Jones, D.; Cunningham, W. Reducing inequalities in care for patients with non-malignant diseases: Insights from a realist evaluation of an integrated palliative care pathway. *Palliat Med* **2016**, *30*, 690-697, doi:10.1177/0269216315626352.

140. Daveson, B.A.; De, W.-L.; Witt, J.; Newson, K.; Morris, C.; Higginson, I.J.; Evans, C.J. Results of a transparent expert consultation on patient and public involvement in palliative care research. *Palliat Med* **2015**, *29*, 939-949, doi:10.1177/0269216315584875.

141. De, K.-V.; M, C.; Pasman, H.R.W.; Schweitzer, B.P.M.; Francke, A.L.; Onwuteaka-Philipsen, B.D.; Deliens, L. General practitioners' perspectives on the avoidability of hospitalizations at the end of life: A mixed-method study. *Palliat Med* **2014**, *28*, 949-958, doi:10.1177/0269216314528742.

142. De, S.; Houttekier, D.; Deliens, L.; Cohen, J. Developing indicators of appropriate and inappropriate end-of-life care in people with Alzheimer’s disease, cancer or chronic obstructive pulmonary disease for population-level administrative databases: A RAND/UCLA appropriateness study. *Palliat Med* **2017**, *31*, 932-945, doi:10.1177/0269216317705099.

143. Forbat, L.; Robinson, R.; Bilton-Simek, R.; Francois, K.; Lewis, M.; Haraldsdottir, E. Distance education methods are useful for delivering education to palliative caregivers: A single-arm trial of an education package (PalliativE Caregivers Education Package). *Palliat Med* **2018**, *32*, 581-588, doi:10.1177/0269216317712849.

144. Goodhead, A.; Speck, P.; Selman, L. 'I think you just learnt as you went along' - Community clergy's experiences of and attitudes towards caring for dying people: A pilot study. *Palliat Med* **2016**, *30*, 674-683, doi:10.1177/0269216315625860.

145. Guo, Q.; Chochinov, H.M.; McClement, S.; Thompson, G.; Hack, T. Development and evaluation of the Dignity Talk question framework for palliative patients and their families: A mixed-methods study. *Palliat Med* **2018**, *32*, 195-205, doi:10.1177/0269216317734696.

146. Hill, H.C.; Paley, J.; Forbat, L. Observations of professional-patient relationships: A mixed-methods study exploring whether familiarity is a condition for nurses' provision of psychosocial support. *Palliat Med* **2014**, *28*, 256-263, doi:10.1177/0269216313499960.

147. Jones, L.; Candy, B.; Davis, S.; Elliott, M.; Gola, A.; Harrington, J.; Kupeli, N.; Lord, K.; Moore, K.; Scott, S., et al. Development of a model for integrated care at the end of life in advanced dementia: A whole systems UK-wide approach. *Palliat Med* **2016**, *30*, 279-295, doi:10.1177/0269216315605447.

148. Jors, K.; Tietgen, S.; Xander, C.; Momm, F.; Becker, G. Tidying rooms and tending hearts: An explorative, mixed-methods study of hospital cleaning staff's experiences with seriously ill and dying patients. *Palliat Med* **2017**, *31*, 63-71, doi:10.1177/0269216316648071.

149. Kimbell, B.; Murray, S.A.; Byrne, H.; Baird, A.; Hayes, P.C.; MacGilchrist, A.; Finucane, A.; Brookes, Y.; O’Carroll, R.E.; Weir, C.J., et al. Palliative care for people with advanced liver disease: A feasibility trial of a supportive care liver nurse specialist. *Palliat Med* **2018**, *32*, 919-929, doi:10.1177/0269216318760441.

150. Latter, S.; Hopkinson, J.B.; Lowson, E.; Hughes, J.A.; Hughes, J.; Duke, S.; Anstey, S.; Bennett, M.I.; May, C.; Smith, P., et al. Supporting carers to manage pain medication in cancer patients at the end of life: A feasibility trial. *Palliat Med* **2018**, *32*, 246-256, doi:10.1177/0269216317715197.

151. Leemans, K.; Deliens, L.; Francke, A.L.; Vander, S.; Van Den, B.; Cohen, J. Quality indicators for palliative care services: Mixed-method study testing for face validity, feasibility, discriminative power and usefulness. *Palliat Med* **2015**, *29*, 71-82, doi:10.1177/0269216314546712.

152. Martins, P.; Hernández-Marrero, P. Palliative care nursing education features more prominently in 2015 than 2005: Results from a nationwide survey and qualitative analysis of curricula. *Palliat Med* **2016**, *30*, 884-888, doi:10.1177/0269216316639794.

153. McIlfatrick, S.; Doherty, L.C.; Murphy, M.; Dixon, L.; Donnelly, P.; McDonald, K.; Fitzsimons, D. ‘The importance of planning for the future’: Burden and unmet needs of caregivers’ in advanced heart failure: A mixed methods study. *Palliat Med* **2018**, *32*, 881-890, doi:10.1177/0269216317743958.

154. McLaughlin, D.; Barr, O.; McIlfatrick, S.; McConkey, R. Developing a best practice model for partnership practice between specialist palliative care and intellectual disability services: A mixed methods study. *Palliat Med* **2014**, *28*, 1213-1221, doi:10.1177/0269216314550373.

155. Michael, N.; O'Callaghan, C.; Brooker, J.E.; Walker, H.; Hiscock, R.; Phillips, D. Introducing a model incorporating early integration of specialist palliative care: A qualitative research study of staff's perspectives. *Palliat Med* **2016**, *30*, 303-312, doi:10.1177/0269216315598069.

156. Pype, P.; Mertens, F.; Wens, J.; Stes, A.; Van Den, E.; Deveugele, M. Preparing palliative home care nurses to act as facilitators for physicians' learning: Evaluation of a training programme. *Palliat Med* **2015**, *29*, 458-463, doi:10.1177/0269216314560391.

157. Reed, E.; Todd, J.; Lawton, S.; Grant, R.; Sadler, C.; Berg, J.; Lucas, C.; Watson, M. A multi-professional educational intervention to improve and sustain respondents’ confidence to deliver palliative care: A mixed-methods study. *Palliat Med* **2018**, *32*, 571-580, doi:10.1177/0269216317709973.

158. Van Den, H.; D, G.M.; Schellekens, M.P.J.; Molema, J.; Speckens, A.E.M.; Van Der, D.; M, A. Mindfulness-Based Stress Reduction for lung cancer patients and their partners: Results of a mixed methods pilot study. *Palliat Med* **2015**, *29*, 652-660, doi:10.1177/0269216315572720.

159. Yardley, I.; Yardley, S.; Williams, H.; Carson-Stevens, A.; Donaldson, L.J. Patient safety in palliative care: A mixed-methods study of reports to a national database of serious incidents. *Palliat Med* **2018**, *32*, 1353-1362, doi:10.1177/0269216318776846.
