# Supplementary material for: A Methodological Review of Mixed Methods Research in Palliative and End-of-Life Care (2014–2019)
Source: Int J Environ Res Public Health. 2020 May 29;17(11):3853. doi: 10.3390/ijerph17113853 (PMC7312170; doi:10.3390/ijerph17113853)
Supplement: Supplementary file 1 [file ijerph-17-03853-s001.zip › Supplementary Material/Supplementary_Material_4.docx]

**Supplementary material 4: Most-cited key literature on mixed methods research**

| **Bibliographic information** | **N** | **%** |
| --- | --- | --- |
| Creswell JW, Clark VLP. *Designing and conducting mixed methods research*, 2nd ed. Thousand Oaks, CA: SAGE, 2007, 2011. | 12 | 26.6 |
| Creswell JW. *Research design: Qualitative, quantitative and mixed method approaches*, 4th ed. Thousand Oaks, CA: SAGE, 2003, 2014 | 6 | 13.3 |
| O’Cathain A, Murphy E and Nicholl J. The quality of mixed methods studies in health services research. *J Health Serv Res Policy* 2008; 13(2): 92–98. | 3 | 6.6 |
| O’Cathain A, Murphy E, and Nicholl J. Three techniques for integrating data in mixed methods studies. *BMJ* 2010; 341:c4587. | 3 | 6.6 |
| Östlund, U., Kidd, L., Wengstrom, Y., et al. Combining qualitative and quantitative research within mixed-method research designs: A methodological review. *Int J Nurs Stud* 2011, 48(3): 369–383 | 3 | 6.6 |
| Tashakkori A and Teddlie C. *Handbook of mixed methods in social and behavioral research*. Thousand Oaks, CA: SAGE, 2003, 2010 | 3 | 6.6 |
| Teddlie C and Tashakkori A. *Foundations of mixed methods research*. Thousand Oaks, CA: SAGE, 2009 | 3 | 6.6 |
| Creswell J, Klassen A, Plano Clark V, and Clegg Smith K. *Best practice for mixed methods research in health science*. Washington, DC: Office of Behavioral and Social Science Research. Web site: [https://www.nursing.virginia.edu/media/Best_Practices_for_ Mixed_Methods_Research.pdf](https://www.nursing.virginia.edu/media/Best_Practices_for_%20Mixed_Methods_Research.pdf) | 2 | 4.4 |
| Creswell J, Plano Clark V, Gutmann M, et al. Advanced mixed methods research designs. In: Tashakkori A and Teddlie C (eds) *Handbook of mixed methods in social and behavioural research*. Thousand Oaks, CA: SAGE, 2010, pp. 209–240 | 2 | 4.4 |
| Farquhar MC, Ewing G and Booth S: Using mixed methods to develop and evaluate complex interventions in palliative care research. *Palliat Med* 2011, 25: 748–757 | 2 | 4.4 |
| Fetters MD, Curry LA and Creswell JW. Achieving integration in mixed methods designs – principles and practices. *Health Serv Res* 2013; 48: 2134–2156 | 2 | 4.4 |
| Guetterman TC and Creswell JW. Integrating quantitative and qualitative results in health science mixed methods research through joint displays. *Ann Fam Med* 2015;13(6):554-561. | 2 | 4.4 |
| Johnson R and Onwuegbuzie A. Mixed methods research: a research paradigm whose time has come. *Educ Res* 2004; 33(7): 14–26. | 2 | 4.4 |
